# Forest carbon sequestration on the west coast, USA: Role of species, productivity, and stockability

**DOI:** 10.1371/journal.pone.0302823

**Published:** 2024-05-31

**Authors:** Paul J. Chisholm, Andrew N. Gray

**Affiliations:** 1 Oak Ridge Institute for Science and USDA Forest Service Pacific Northwest Research Station, Corvallis, Oregon, United States of America; 2 USDA Forest Service Pacific Northwest Research Station, Corvallis, Oregon, United States of America; Feroze Gandhi Degree College, INDIA

## Abstract

Forest ecosystems store large amounts of carbon and can be important sources, or sinks, of the atmospheric carbon dioxide that is contributing to global warming. Understanding the carbon storage potential of different forests and their response to management and disturbance events are fundamental to developing policies and scenarios to partially offset greenhouse gas emissions. Projections of live tree carbon accumulation are handled differently in different models, with inconsistent results. We developed growth-and-yield style models to predict stand-level live tree carbon density as a function of stand age in all vegetation types of the coastal Pacific region, US (California, Oregon, and Washington), from 7,523 national forest inventory plots. We incorporated site productivity and stockability within the Chapman-Richards equation and tested whether intensively managed private forests behaved differently from less managed public forests. We found that the best models incorporated stockability in the equation term controlling stand carrying capacity, and site productivity in the equation terms controlling the growth rate and shape of the curve. RMSEs ranged from 10 to 137 Mg C/ha for different vegetation types. There was not a significant effect of ownership over the standard industrial rotation length (~50 yrs) for the productive Douglas-fir/western hemlock zone, indicating that differences in stockability and productivity captured much of the variation attributed to management intensity. Our models suggest that doubling the rotation length on these intensively managed lands from 35 to 70 years would result in 2.35 times more live tree carbon stored on the landscape. These findings are at odds with some studies that have projected higher carbon densities with stand age for the same vegetation types, and have not found an increase in yields (on an annual basis) with longer rotations. We suspect that differences are primarily due to the application of yield curves developed from fully-stocked, undisturbed, single-species, “normal” stands without accounting for the substantial proportion of forests that don’t meet those assumptions. The carbon accumulation curves developed here can be applied directly in growth-and-yield style projection models, and used to validate the predictions of ecophysiological, cohort, or single-tree style models being used to project carbon futures for forests in the region. Our approach may prove useful for developing robust models in other forest types.

## 1. Introduction

Atmospheric carbon dioxide has increased at an accelerating rate in recent decades and its effects on climate, and climate impacts on human populations, are well-established [1]. While emissions from combustion of fossil fuels are a leading cause of global warming, land use can also be a significant source, or sink, of carbon. Temperate forests have the potential to store significant amounts of terrestrial carbon [2, 3]. For example, forestland in the United States accumulated 615 Tg CO_2_e/yr in 2020, offsetting 11% of greenhouse gas emissions in the country [4]. The Pacific coastal forests of the United States are among the most carbon-dense ecosystems on the planet [5, 6], capable of storing up to 4595 Mg of aboveground live biomass per hectare [7]. The governments of Pacific coast states (Washington, Oregon, and California) have recently shown an interest in incentivizing forest carbon storage above current levels [8].

Determining the carbon storage potential of forests and their response to different types of management requires the use of projection models. Live trees are often the largest pool of forest carbon after mineral soil [9, 10], but the live tree pool is often more dynamic and is the primary source of carbon cycling in the rest of the ecosystem. As such, estimating rates of carbon sequestration in live trees is important for land managers seeking to maximize carbon storage on the landscape and/or in harvested wood products. A variety of projection models have been used to estimate future carbon stores, including ecosystem process models [11–13], landscape process models [14], and individual tree simulators [15]. The CBM-CFS3 model [16] is another forest sector systems model driven primarily by growth-and-yield curves that has become the primary carbon accounting tool for international reporting for Canada, has been applied in several US states (e.g., [17]), and is being developed for application in the Pacific coast states.

The development of accurate growth and yield models describing the rate of live tree carbon accumulation with stand age in various forested ecosystems is necessary for applying the CBM-CFS3 model and critical for evaluating the behavior of other projection models. Although growth and yield models for merchantable tree volume currently exist for many Pacific coast ecosystems (e.g., [18, 19]), the deployment of these models for regional assessment is hampered by a number of factors. First, models are often limited by geography, ownership, or forest type. Many models are fit to data collected on either public or private land in specific regions, and it is unclear how well these models may perform in stands under different ownership since productivity and management intensity often varies across ownerships [20, 21]. Models applied to different forest types may also be derived from many different studies that may employ inconsistent or opaque methodology [22–24], making consistent regional assessments difficult. Finally, plot locations used to establish yield curves may not always be truly random [25, 26], and instead be biased towards “ideal” forested stands—the so-called “majestic forest bias” [23, 24, 27]. Other models have been developed for highly managed stands, such as plantations [28], and are not suitable for broad landscape-scale application. In most cases, yield tables are intentionally developed for “normal” stands that are fully-stocked with free-growing trees. Stands that fall in transition zones, disease pockets, along habitat edges, shallow or droughty soils, or that contain non-stockable openings such as cliffs and scree fields are under-represented in the literature [24, 29]. If models are to be implemented at a landscape scale, then these types of stands should either be included in any modelling dataset in proportion to their abundance on the landscape, or variables that account for these conditions need to be included in the modeling framework (e.g., [30]).

National forest inventory data have the potential to address many of these issues. In the United States, the US Forest Service’s Forest Inventory and Analysis (FIA) program systematically samples all forested lands regardless of ownership, productivity, protected status, or current condition [31]. In addition to individual tree measurement and remeasurement, measurement of site index trees, tree age, topography, potential stocking, and plant association provide potentially useful variables for growth and yield model development. National forest inventory data has been similarly used to develop growth and yield models in other countries, such as Finland [32].

Since forests often accumulate biomass over a period of many centuries, formulating accurate yield curves based on observed growth can be a challenging and prolonged process, especially in less productive ecosystems. Yield curves based on repeated measurement of forested plots may take many decades to develop (e.g. [33]). Consequently, yield curves are often formulated based on a chronosequence, in which multiple forests at different stages of development are measured concurrently and grouped by site productivity class. However, developing yield curves from chronosequences requires assumptions which are often difficult to evaluate [34, 35]. Foremost among these is the assumption that all samples within a particular chronosequence are identical except for age [34]. This requires the modeler to carefully stratify the sample population so that biologically similar samples are placed within the same pool. Within forest growth and yield modeling, several parameters have been implemented to define these stratifications such as species composition, climate, topography, soil, and productivity.

One of the major challenges to the development of accurate growth and yield models is differing management regimes across various ownerships. Within the Pacific coast region, forests are owned by a variety of federal, state, and local government agencies, in addition to private individuals, non-profit organizations, Native tribes, and industrial timber operations, among others. Differences in the average production of merchantable timber volume, use of prescribed fire, and treatments to enhance habitat or promote large tree growth may be observed among ownership groups as a consequence of differing management goals.

Industrial timberland, for instance, is typically managed for merchantable biomass production, while various public and non-industrial private owners may have other management goals that take precedence over biomass production. However, determining the direct influence of these factors on yield curves can be problematic in observational datasets due to confounding variables such as productivity and stockability. Industrial timber interests have typically selected more innately productive forest land throughout the Pacific Northwest, so it is no surprise that yields for industrially-owned timberland are higher than for other ownerships, such as the US Forest Service. What is unclear is how much of that difference is due to the inherent productivity of the land selected by industrial timber interests, and how much is due to differing management regimes. A single model, based on functional traits and capable of describing biomass trajectories across multiple owner groups, would be a significant improvement over a system in which different owner groups require different models.

In this paper, we formulate a biomass yield model to apply to the full range of existing forests based on two functional traits—stand stockability and productivity—within an ecological characterization of vegetation types for all forests in the Pacific coast region. We use the model to fit FIA data from two of the largest owner classes, corporate timber interests and the US Forest Service, to test if the yield curves are significantly different. We then provide an example application of the model, predicting how live biomass in one of the more economically important vegetation types would be affected under different rotation lengths.

## 2. Methods

### 2.1 Study area

The study area consists of the forested lands within the U.S. states of California, Oregon, and Washington, which range from 32.6–49.0 degrees N Latitude, -124.7 –-114.2 degrees W Longitude, 0–3570 m elevation, 54–6100 mm mean annual precipitation, and 0.8–25.1 degrees mean annual temperature (climate from [36]). Summers are typically dry, with precipitation delivered in synoptic storms from the Pacific Ocean. Annual precipitation is highest in the Coast Ranges, Cascades, and Sierra Nevada mountain ranges, and lowest in the lower elevations to the east of these ranges. Forest vegetation varies from pinyon-juniper and oak woodlands in the driest areas (*Pinus*, *Juniperus*, and *Quercus* spp., respectively); to Sitka spruce, western hemlock, and coast redwood in the wetter areas near the ocean (*Picea sitchensis*, *Tsuga heterophylla*, and *Sequoia sempervirens*); to mountain hemlock, subalpine fir and Engelmann spruce in the coldest areas with abundant snowpack (*Tsuga mertensiana*, *Abies lasiocarpa*, and *Picea engelmanii*). Taxonomy follows USDA NRCS [37]. The most abundant species are Douglas fir (*Pseudotsuga menziesii*) and Ponderosa pine (*Pinus ponderosa*), well-distributed in the moist-mesic and dry-mesic portions of the region, respectively [38, 39].

Of the 33.7 million ha of forestland in the region, 61% is in public ownership, and 16% of the total is designated as reserved from management for the production of timber products [40]. Non-reserved public lands are managed for a variety of values, including timber, wildlife habitat, water production, and resource conservation. Of the 13.1 million ha of private forestland, 5.1 million ha is managed by corporate owners, most of which is managed to maximize net present value of timber products.

### 2.2 Inventory design

Because the inventory covers all forested land regardless of ownership, FIA data provide an excellent opportunity to develop growth and yield models that are appropriate for landscape-level application. The inventory sample consists of a randomized-systematic grid of points at a sample density of one per ~2,400 ha, which is intensified on Pacific Northwest Region National Forest lands outside designated Wilderness areas to a sample density of one per ~750 ha. Field plots are installed at every location containing accessible forest land. Under the FIA definition, trees must currently or recently constitute at least 10% canopy cover in a contiguous area greater than 0.4 ha in size, and not be subject to a nonforest land use (e.g., mowing or pasture) to qualify as forest. The FIA plot design consists of nested fixed-radius subplots around four points of 2.1 m radius for trees > 2.5 cm diameter at breast height (DBH), 7.3 m radius for trees > 12.7 cm DBH, and 18 m radius for trees > 76 cm DBH in western Oregon and Washington, and for trees ≥ 61 cm DBH elsewhere in the region. Each tree is measured for diameter, height, species, and several other attributes.

Data were drawn from the most recent available measurement cycle of the annual-design inventory, which ran from 2010 to 2019. We used conditions as our unit of replication. A condition in the FIA dataset represents a stand of generally uniform characteristics, including ownership, stand size, forest type, and reserved status. A single plot, because of its systematic location and design, can have multiple conditions. Consequently, each data point in our analysis constitutes a single condition, which may include an entire FIA plot or only a portion of a plot. For our models, we only used forested conditions that occupied at least 75% of a plot (~0.3 ha). This ensured that the sampled area was sufficiently large to accurately estimate stand-level attributes such as live biomass per acre. FIA crews assign a stand age to each forested condition based on the average age of the overstory trees, usually determined by increment-coring a subsample of trees. The timing and type of disturbances and treatments occurring prior to measurement were recorded on each plot based on information from prior measurements, landowner records, and/or evidence on the plot; on the west coast these records included all significant events affecting the current stand.

### 2.3 Data selection

The goal of this study was to accurately describe the rate of biomass accumulation in undisturbed forested stands. Many FIA plots have experienced natural disturbances such as fire, or human-caused treatments such as thinning. We excluded stands from our analysis that had been disturbed by these agents. Within the study area, FIA crews recorded all discernible treatments and disturbances affecting the current stand for each forested condition, regardless of how long ago it may have occurred. These disturbance/treatment designations were made based on a combination of visual evidence on the plot, records of past visits by inventory crews, or landowner records.

Stands were determined to be undisturbed if they did not experience a human-caused treatment (such as thinning) since stand origin. Fires that occurred since stand origin were allowed as long as they occurred more than 100 years before plot measurement. Because we wanted to preserve some level of background mortality, we did not exclude stands that had been disturbed by other agents, such as insects or disease.

### 2.4 Calculating plot biomass

We calculated biomass (above and belowground) for each live tree using the compilation approach used for regional carbon assessments (e.g., [10]). Bole biomass (ground to tip) was calculated from regional species-specific volume equations documented in Zhou and Hemstrom [41] and species-specific wood density values documented in Woudenberg et al. [42]. Bark and branch biomass were calculated from regional species-specific equations selected from Means et al. [43] and documented in Zhou and Hemstrom [41], except branch Eq 16 used Snell and Little [44] and bark Eqs 8 and 20 used Means et al. [43] Eqs 5 and 275, respectively. Most equations use both DBH and height data, whereas a few bark and branch equations use DBH only. Foliage and belowground (coarse roots > 2 mm diameter) biomass were calculated using the Jenkins et al. [45] ratios to total tree biomass as implemented in Woodall et al. [46], which adjusts the estimate by the ratio of the FIA volume-based estimate of bole biomass to the Jenkins equation-based estimate of bole biomass. Calculated tree biomass was expanded to a hectare basis using the relevant sample plot size. For each condition, live tree biomass values (Mg ha^-1^) were summed and divided by the proportion of the plot covered by the condition.

### 2.5 Independent variables

We compared two methods of classifying the landscape for modeling: by current forest type, and by climax plant association zone (PAZ). FIA calculates current forest type based on the tree species representing the plurality of stocking of overstory trees at the time of measurement, which are then aggregated into forest type groups. In contrast, the PAZ describes the tree and understory plant species that will become dominant as the stand approaches maturity [47] (Table 1). The PAZ classes tend to better reflect site conditions than the dominance by ubiquitous, long-lived early seral species common to the region (e.g., [48]). PAZ classes were assigned using a hierarchical key based on cover of tree and indicator understory species. The PAZ classification is the result of a multi-year effort by the US Forest Service Pacific Northwest Region Ecology Program that draws on local forest succession research (e.g., [49, 50]).

**Table 1:** Study area climax Plant Association Zones (PAZ).

Vegetation zone	Sample size	Dominant tree species
Douglas Fir	747	*Pseudotsuga menziesii*, *Pinus ponderosa*, *Quercus chrysolepis*
Pinyon-Juniper	353	*Juniperus occidentalis*, *Juniperus californica*, *Pinus monophylla*
Foothill Pine-Coulter Pine	71	*Quercus douglasii*, *Pinus sabiniana*, *Quercus wislizeni*
Hardwoods	113	*Quercus garryana*, *Populus fremontii*, *Quercus douglasii*
Jeffrey Pine	106	*Pinus jeffreyi*, *Juniperus occidentalis*, *Pinus ponderosa*
Lodgepole Pine	42	*Pinus contorta*, *Pinus radiata*, *Quercus agrifolia*
Mountain Hemlock	512	*Tsuga mertensiana*, *Abies amabilis*, *Pseudotsuga menziesii*
Parklands	327	*Pinus contorta*, *Abies lasiocarpa*, *Tsuga mertensiana*
Ponderosa Pine	465	*Pinus ponderosa*, *Quercus garryana*, *Juniperus occidentalis*
Port Orford Cedar	20	*Pseudotsuga menziesii*, *Chamaecyparis lawsoniana*, *Lithocarpus densiflorus*
Red Fir	122	*Abies magnifica*, *Pinus contorta*, *Abies shastensis*
Redwood	63	*Sequoia sempervirens*, *Pseudotsuga menziesii*, *Lithocarpus densiflorus*
Silver Fir	786	*Tsuga heterophylla*, *Pseudotsuga menziesii*, *Abies amabilis*
Sitka Spruce	193	*Tsuga heterophylla*, *Pseudotsuga menziesii*, *Thuja plicata*
Subalpine Fir-Engelmann Spruce	310	*Abies lasiocarpa*, *Pinus contorta*, *Pseudotsuga menziesii*
Tanoak	211	*Pseudotsuga menziesii*, *Lithocarpus densiflorus*, *Arbutus menziesii*
Western Hemlock	1767	*Pseudotsuga menziesii*, *Tsuga heterophylla*, *Thuja plicata*
Western Red Cedar	43	*Pseudotsuga menziesii*, *Thuja plicata*, *Larix occidentalis*
White Fir-Grand Fir	1272	*Abies concolor*, *Pseudotsuga menziesii*, *Pinus ponderosa*
Total	7523	

For each PAZ, plot counts and the three most abundant tree species by basal area are listed in descending order.

In addition to analyzing by forest type group or PAZ, our models incorporated metrics for stockability and productivity. Productivity was estimated using the mean annual increment at culmination for the stand (MAI, m^3^ha^-1^yr^-1^). This metric is calculated from site tree data gathered by field crews inserted into species-appropriate age:height models [51]. Stockability can be influenced by non-stockable ground surfaces, such as rubble fields, bogs, and stable shrub patches, as well as climatic and soil conditions that limit trees from fully occupying a site. Stockability was estimated using live plus missing canopy cover, an estimate made by field crews using evidence of live and dead trees which represents the theoretical maximum canopy cover (MCC) the stand can support in the absence of disturbances and treatments. These variables are appropriate for forest chronosequence data because they are static and do not change as the stand matures.

### 2.6 Modeling

Model fitting and data manipulation were conducted using R version 4.1.2 within RStudio version 2021.09.0. Data were fit to models with nonlinear least squares using the nls() function from the stats() R package. Models were based on the Chapman-Richards equation, taking the form:

$$y(t)=ymax{\left(1-e^{-kt}\right)}^{p}$$ (1)

where *y(t)* equals total live tree biomass (Mg/ha) at time *t*, *t* equals the stand age (in years), *ymax* is the carrying capacity of the stand (Mg ha^-1^), *k* controls the intrinsic growth rate, and *p* controls the shape of the response. Data were heteroskedastic, with variance in biomass increasing with stand age. Consequently, model fits implemented weights equal to the inverse of the stand age to account for nonconstant variance.

### 2.7 Grouping by forest type or plant association zone

To determine whether the data were better described by current forest type group or PAZ, data were fit to the Chapman-Richards equation using either grouping. Model fits were compared with AIC. This analysis included 17 different forest type groups (S1 Table in S1 File) and 19 PAZs (Table 1). Separate models were fit to each forest type or PAZ using a binary dummy variable (*FT* or *PAZ* in the equations below):

$$y(t)={PAZ}_{1}\times {ymax}_{1}{\left(1-e^{-k_{1}t}\right)}^{p_{1}}+\cdots +{PAZ}_{19}\times {ymax}_{19}{\left(1-e^{-k_{19}t}\right)}^{p_{19}}$$ (2)

$$y(t)={FT}_{1}\times {ymax}_{1}{\left(1-e^{-k_{1}t}\right)}^{p_{1}}+\cdots +{FT}_{17}\times {ymax}_{17}{\left(1-e^{-k_{17}t}\right)}^{p_{17}}$$ (3)

We also examined past measurement data to determine how frequently forest type changes in a stand between measurement periods.

### 2.8 Incorporating productivity and stockability

Variables estimating productivity (mean annual increment at culmination, MAI) and stockability (maximum canopy cover, MCC) were incorporated into the model through direct modification of the three Chapman-Richards model parameters *ymax*, *k*, and *p*. Each of the Chapman-Richards model parameters were transformed to be either a linear or exponential response of the variable of interest—i.e., ymax=a×MCC or $ymax=a\times {MCC}^{b}$. Modifying a parameter to incorporate both variables (for example, $a\times {MAI}^{b}\times {MCC}^{c}$) resulted in unstable model convergence; consequently, each parameter was modified with only one variable (either MAI or MCC) to avoid this issue. As such, each of the three Chapman-Richards parameters had five different possible configurations for a total of 5^3^ = 125 unique possible models. Each model applied the same form of the equation to each of the 19 PAZs. Models were evaluated using AIC.

### 2.9 Non-zero intercept

During preliminary data inspection it became apparent that many relatively young stands had disproportionately high concentrations of live biomass. This is due either to the uneven-aged nature of many of the plots, or the presence of surviving remnant trees from past harvests or disturbances at insufficient density to define the stand age. However, the basic Chapman-Richards equation does not allow for a non-zero y-intercept, so we modified the equation with an additive constant, *c*. The modified equation took the form

$$y(t)=ymax{\left(1-e^{-kt}\right)}^{p}+c$$ (4)

where each of the base Chapman-Richards parameters were modified according to the best model identified in section 2.8. This modified version of the Chapman-Richards equation was compared to the original form of the model using AIC to determine if the addition of a non-zero intercept significantly improved model fit.

### 2.10 Ownership

To determine if model adjustments for stockability and productivity adequately accounted for differences between corporate and Forest Service-owned stands within a PAZ, we selected 3 PAZs (western hemlock, white fir-grand fir, and Douglas fir) that had adequate representation from both corporate and Forest Service ownership (*N* > 75 plots per category) and fit models to stands from those ownership classes (S2 Table in S1 File). These ownership classes were selected for comparison because they had sufficient representation in the dataset and we expected them to follow different biomass accumulation trajectories. We used a binary dummy variable to fit separate models to each ownership class within each PAZ. Each PAZ was modelled separately. This modified model grouping the data by ownership class was compared to the null model with an F-test to determine if the inclusion of ownership significantly improved model fit. The F-test used the F-distribution to compare the residual sum of squares of the null model to the residual sum of squares of the model grouping the data by ownership and determine if the improvement in model fit justified the additional degrees of freedom of the model grouping the data by ownership. These comparisons (grouped by ownership vs. no grouping by ownership) were made first in the context of the simple model, which fit data to a basic Chapman-Richards curve without adjusting for productivity or stockability, and then with the “best” model identified in section 2.8 which incorporated stockability and productivity. This comparison allowed us to determine whether the curve for corporate stands was significantly different from the curve for Forest Service stands within a single PAZ, both before and after the incorporation of stockability/productivity into the model. This would help us determine if the same model could be applied to stands from both ownership groups once adjustments are made for productivity and stockability, or if separate models are needed.

### 2.11 Rotation length and biomass storage

As an example application of our models, we sought to determine how much standing live biomass would exist on the landscape under different rotation regimes on corporate land in the western hemlock zone, one of the most productive and economically important vegetation types with a large sample size in multiple ownerships. We tested five different rotation lengths (35, 40, 50, 60, and 70 years) under the assumption of a fully regulated forest where stands would be divided equally among ages from 0 to the rotation length. Current rotation ages for corporately-owned timberland in the western hemlock zone range roughly from 35 to 45 years. Total corporate timberland in the western hemlock zone was estimated to be 2,029,445 hectares using standard FIA post-stratification estimators [31, 42]. We used the model for the western hemlock zone to predict the biomass (Mg/ha) for each stand age. We then multiplied this by the estimated land area within each age cohort (2,029,445 divided by the number of years in the rotation length). For model inputs, we used the median values for productivity (11.31 m^3^ ha^-1^ yr^-1^) and stockability (90% maximum canopy cover) from corporate plots within the western hemlock zone. These values are slightly higher than the values for the western hemlock zone as a whole (S1 Fig in S1 File).

## 3. Results

### 3.1 Selected equation form

During initial screening of the data, 7523 stands were determined to fit the criteria for inclusion in the analysis. Grouping the data by PAZ (model 2) was a better fit than grouping by current forest type (model 3, S3 Table in S1 File). Additionally, inventory remeasurement data revealed that over the ~10-yr remeasurement period, 11.8% of stands experienced a change in forest type.

Stand stockability and productivity were incorporated into the model in 125 different combinations. The best performing model (Table 2) incorporated stockability as an exponential modifier of *ymax*, and productivity as an exponential modifier of *k* and *p*, taking the form

$$y(t)=a_{1}{MCC}^{b1}{\left(1-e^{-{a_{2}MAI}^{b2}t}\right)}^{{a_{3}MAI}^{b3}}$$ (5)

**Table 2:** AIC values for the top 10 tested models.

	Parameter form			
Model rank	*YMAX* =	*k* =	*p* =	AIC
1	a_1_MCC^b1^	a_2_MAI^b2^	a_3_MAI^b3^	84265
2	a_1_MCC	a_2_MAI^b2^	a_3_MAI^b3^	84276
3	a_1_MCC^b1^	a_2_MAI	a_3_MAI^b3^	84299
4	a_1_MCC	a_2_MAI	a_3_MAI^b3^	84317
5	a_1_MAI^b1^	a_2_MCC^b2^	a_3_MCC^b3^	84359
6	a_1_MCC^b1^	a_2_MAI^b2^	a_3_MCC^b3^	84373
7	a_1_MAI^b1^	a_2_MCC^b2^	a_3_MAI^b3^	84377
8	a_1_MCC^b1^	a_2_MAI^b2^	p	84390
9	a_1_MAI^b1^	a_2_MCC^b2^	p	84391
10	a_1_MCC	a_2_MAI^b2^	a_3_MCC^b3^	84397

Different models were tested which employed various modifications to the base parameters of the Chapman-Richards equation (Eq 1). Each of the three base Chapman-Richards parameters was modified by linear or exponential derivatives of either stockability (estimated by maximum canopy cover, *MCC*) or productivity (estimated by the mean annual increment at culmination, *MAI*), or unmodified. In the best model, stockability modified the upper asymptote, or carrying capacity, of the yield curve, while productivity modified the intrinsic growth rate *k* and the shape *p* of the curve.

The best-performing model incorporated both stockability and productivity (Table 2). However, among the models that incorporated only one of these modifiers, the best form of the model incorporating stockability only (*AIC* = 85453) was a better fit than the best form of the model incorporating productivity only (*AIC* = 85722).

Modifying the Chapman-Richards to incorporate a non-zero intercept resulted in a better model fit (*AIC* = 84222) over the original model (*AIC* = 84265) that did not allow for a non-zero intercept. Thus, the final model for each individual PAZ took the form

$$y(t)=a_{1}{MCC}^{b1}{\left(1-e^{-{a_{2}MAI}^{b2}t}\right)}^{{a_{3}MAI}^{b3}}+c$$ (6)

Expanded to each of the 19 PAZs, the model took the form

$$y(t)={PAZ}_{1}(a_{1.1}{MCC}^{b1.1}{\left(1-e^{-{a_{2.1}MAI}^{b2.1}t}\right)}^{{a_{3.1}MAI}^{b3.1}}+c_{1})+\cdots +{PAZ}_{19}(a_{1.19}{MCC}^{b1.19}{\left(1-e^{-{a_{2.19}MAI}^{b2.19}t}\right)}^{{a_{3.19}MAI}^{b3.19}}+c_{19})$$ (7)

**Fig 1:**
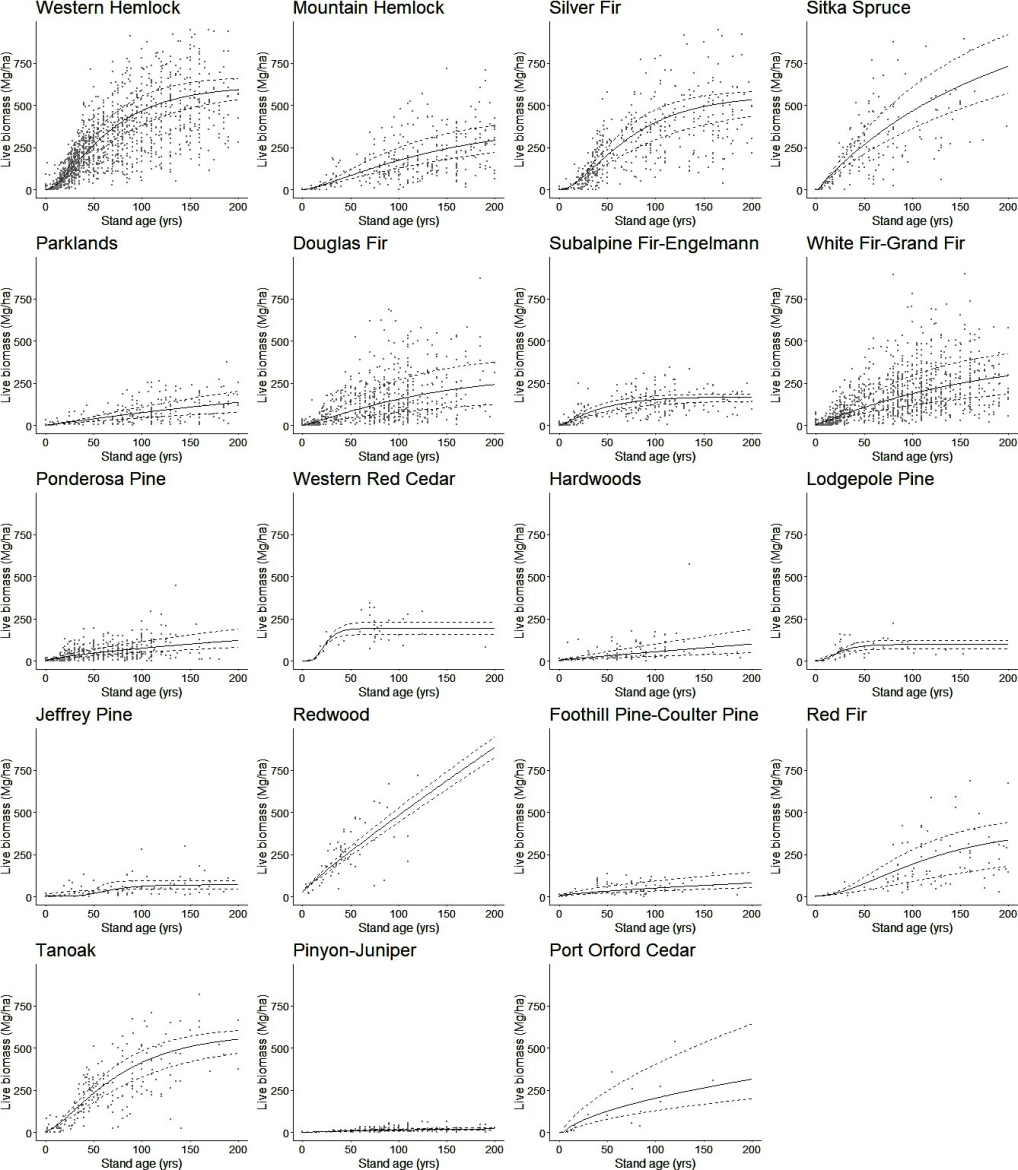
Biomass yield curves for each vegetation zone, using the modified form of the Chapman-Richards equation that performed best in model comparison (6). Biomass figures include all live tree biomass from the bole, branches, bark, and coarse roots. Under this form of the model, stockability determines the upper asymptote for each vegetation zone, while productivity determines the growth rate and shape. This form of the model includes a non-zero y-intercept. The solid line depicts the curve produced by using median values for productivity and stockability within each zone, while the two dashed lines depict the upper and lower quartiles for productivity and stockability within each zone.

Final model coefficients are shown in Table 3. Predicted response curves demonstrate a diversity of shapes and biomass accumulation rates among PAZs (Fig 1). Live biomass increased rapidly with stand age and was still increasing at age 200 in the most productive PAZs (e.g., western hemlock, Sitka spruce, Tanoak). Biomass increased slowly and in some cases leveled off with age in the less productive PAZs (e.g., subalpine fir-Engelmann spruce, hardwoods, and lodgepole pine).

**Table 3:** Coefficients for the best model.

	Model coefficients							RMSE
PAZ	a1	a2	a3	b1	b2	b3	c	(m2/ha)
Mountain Hemlock	22.3	0.00194	0.724	0.683	1.01	0.432	0.063	101
Subalpine Fir-Engelmann Spruce	3.89	0.0825	6.19	0.855	-0.0375	-0.297	2.68	57
Hardwoods	0.0449	0.00196	2.2E-5	1.78	2.64	10.9	2.73	54
Western Hemlock	8.27	0.00188	0.727	0.966	1.03	0.407	3.01	125
Ponderosa Pine	21.4	0.000169	0.6	0.944	0.814	0.153	1.31	34
Parklands	13	0.00179	0.828	0.791	0.695	0.273	2	57
White Fir-Grand Fir	9.42	0.000457	0.385	0.891	1.62	0.65	6.4	96
Silver Fir	4.8	0.00235	0.502	1.07	0.968	0.657	1.21	137
Douglas Fir	1.06	0.00208	0.781	1.35	1.05	0.408	2.56	79
Sitka Spruce	3.89	0.000426	0.274	1.18	1.33	0.623	-4.09	133
Western Red Cedar	0.185	0.148	3.05	1.6	-0.138	0.631	2.4	52
Lodgepole Pine	2.6	1.21	11.4E4	0.871	-1.48	-6.02	0.751	38
Jeffrey Pine	1.68	0.00182	0.0115	1.02	2.44	4.74	2.83	39
Redwood	241	0.000266	1.34	1.1	-0.516	-0.175	9.22	122
Foothill Pine-Coulter Pine	1.08	0.0152	1.96	1.02	0.933	0.0221	3.99	35
Red Fir	9.27	0.00333	1.38	0.943	0.684	0.192	1.11	97
Tanoak	2.25	0.00371	1.02	1.23	0.773	0.264	10.8	109
Pinyon-Juniper	2.18	0.00696	0.845	0.817	0.27	0.271	0.252	10
Port Orford Cedar	65.1	0.0000547	0.479	0.714	1.62	0.187	-28.2	37

Coefficients and root mean square error (RMSE) for the best overall model as determined by AIC, for each climax plant association zone (PAZ). The final model took the form $y(t)=a_{1}{MCC}^{b1}{\left(1-e^{-{a_{2}MAI}^{b2}t}\right)}^{{a_{3}MAI}^{b3}}+c$

### 3.2 Ownership

When using the simple model (2) that did not adjust for stockability or productivity, fitting separate models by ownership class (corporate vs. Forest Service) improved model fit within the western hemlock (*F*_4,1305_ = 19.9, *P* < 0.0001), white fir-grand fir (*F*_4,1153_ = 2.34, *P* = 0.05), and Douglas fir (*F*_4,549_ = 4.93, *P* = 0.0007) vegetation zones. Under the complex model (5) incorporating productivity and stockability, ownership no longer had a significant impact in the white fir-grand fir (*F*_7,1150_ = 1.44, *P* = 0.18) and Douglas fir (*F*_7,546_ = 1.46, *P* = 0.18) zones. Ownership remained a significant factor in the western hemlock zone (*F*_7,1302_ = 6.09, *P* < 0.0001).

**Fig 2:**
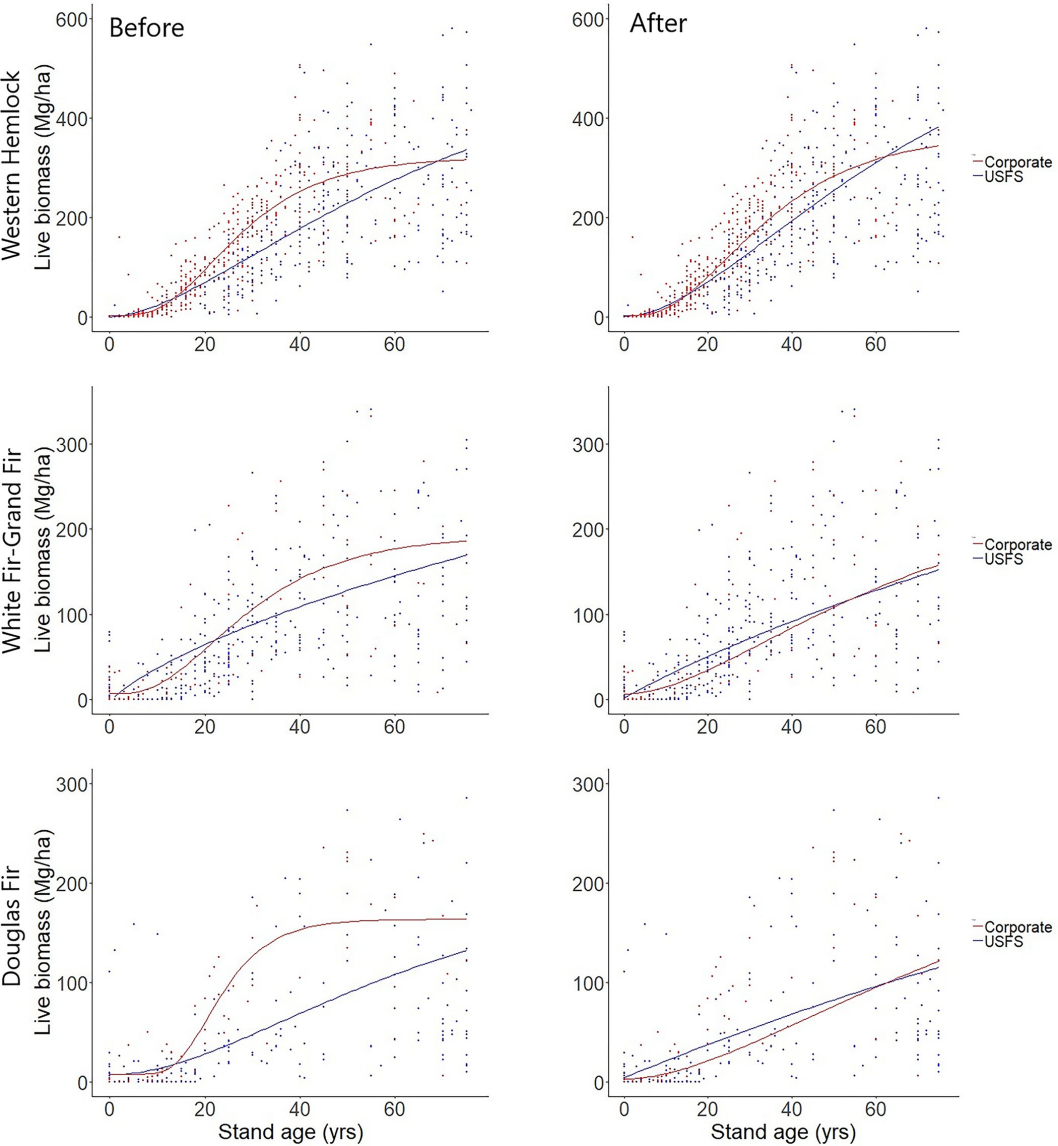
Biomass yield curves for corporate vs. Forest Service-owned stands, before and after adjusting for productivity and stockability. Within each PAZ, a separate model was fit to each owner class using the simple version of the Chapman-Richards equation and the complex version incorporating productivity and stockability. Although there were significant differences between the two curves in the unadjusted model, there were no significant differences between the two curves after adjusting for productivity and stockability.

Much of the difference between the corporate and USFS curves in the western hemlock PAZ was driven by deviations beyond the standard rotation age of the region (Fig 2). Although both curves maintained a similar trajectory until ~50 years of age, the corporate curve plateaued at ~50 years while the Forest Service curve continued to accumulate biomass. Consequently, we re-ran the comparison using only stands that were below the 90% percentile cutoff for stand age within the corporate ownership class (50 years old). When the sample was restricted to stands less than 50 years old, the corporate curve was still significantly different from the Forest Service curve in the unadjusted model (*F*_4,580_ = 18.6, *P* < 0.0001), but not significantly different (*F*_7,577_ = 1.94, *P* = 0.062) when using the complex model incorporating stockability and productivity.

### 3.3 Effect of rotation length on standing live biomass

**Fig 3:**
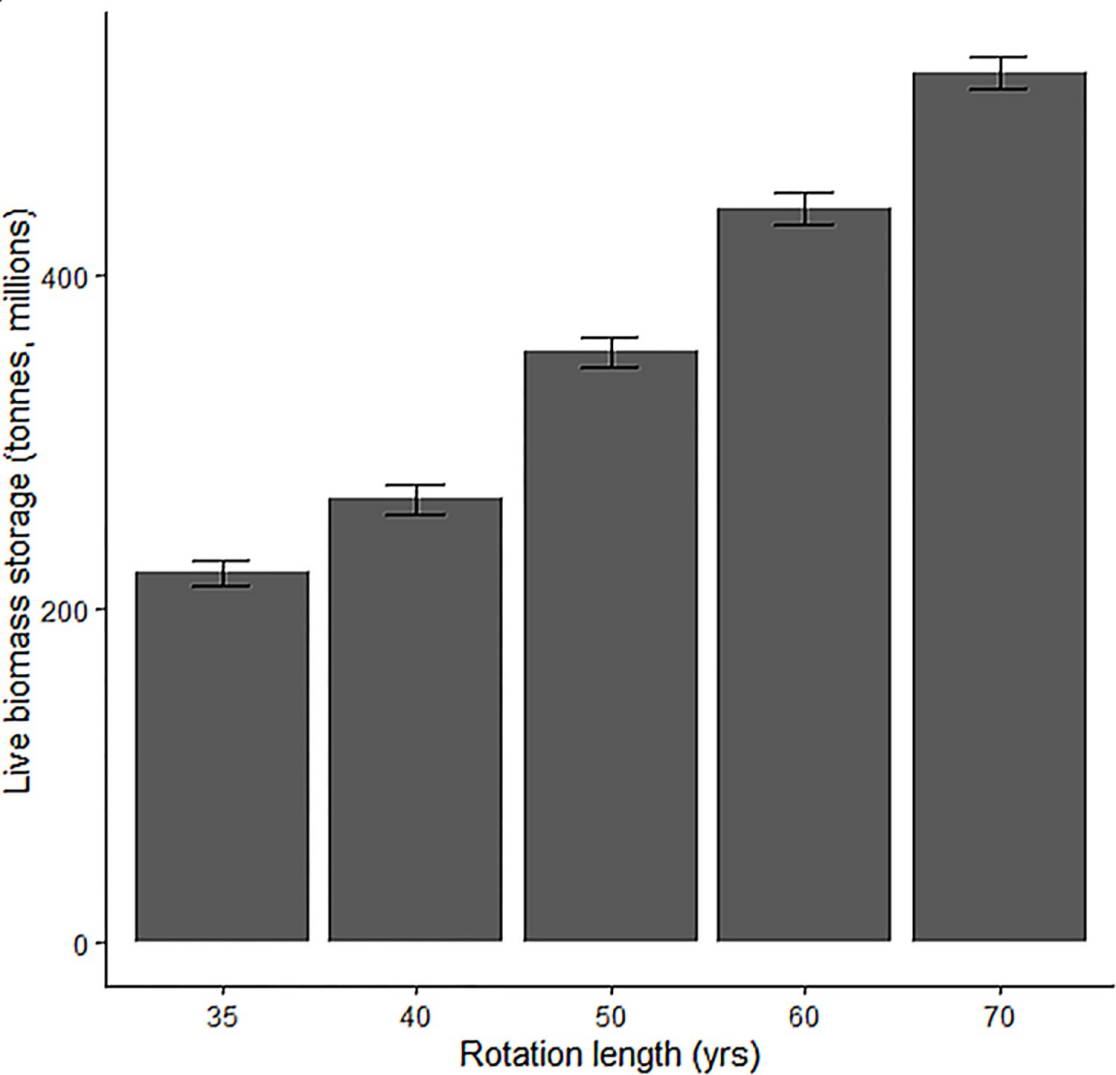
Estimated live biomass storage under different rotation regimes on corporate timberland in the western hemlock zone, assuming an even distribution of stand ages on 2,029,445 hectares of land. Predictions used the model from the western hemlock zone using the median values for productivity and stockability for all corporately-owned plots within the western hemlock zone. Error bars depict the variance of the modelled estimate.

As an example application of our models, we sought to determine how much standing live biomass would exist on the landscape under different rotation regimes on corporate land in the western hemlock zone. Moving from a 40 year rotation to a 50 year rotation, this analysis estimates that an additional 70.5 million metric tons of live biomass (~35.2 million metric tons of carbon) would be stored within corporate timberland in the western hemlock zone. A 70-year rotation would store 414.7 million metric tons of biomass, compared to 176.1 million metric tons of biomass under a 35-year rotation; a 2.35 times increase in biomass for twice the rotation length (Fig 3).

## 4. Discussion

Our study is the first to develop empirical predictions of live biomass accumulation with stand age for all forested vegetation types on the west coast that incorporate metrics for productivity and stockability. These should be useful alternatives to the variety of yield models fit to single-species, fully-stocked, timber-producing forests in selected geographic areas. Other studies have modelled forest types within this region, including mixed conifer stands in the Sierra Nevada [52, 53] and California Coast Range [54], coastal stands of pure Douglas-fir [55–57], coastal mixed-conifer forest [58, 59], eastside ponderosa pine [60, 61], and less common forest types such as poplar plantations [62], among many others. However, nearly all these model sets are based on data from highly managed stands that eventually achieved full stocking. Low-productivity, poorly stocked, and unmanaged forests are largely absent from the growth and yield literature for this region. Consequently, deploying models fit to idealized forested stands within landscape-scale carbon sequestration models could result in a significant overestimation of regional stocks of forest carbon. To our knowledge, our study is the first to create a model flexible enough to be applied at a landscape scale to any forest type within this region.

We used estimated maximum canopy cover as a proxy for stockability, and average merchantable volume increment at culmination as a proxy for productivity. Both variables proved to be important contributors to the model. The best model is biologically reasonable, in that stockability exponentially modified the carrying capacity, and productivity exponentially modified the growth rate and shape of the curve. Model fit was significantly reduced when the model was restricted to only one of the variable modifiers. However, among the models that only incorporated one modifier, the best model incorporating stockability only was a better fit than the model incorporating productivity only. This is probably because the plant association zone classification captures gross differences in productivity (S1 Fig in S1 File) so that within a zone, stockability is a better predictor of biomass accumulation than productivity.

Although many other studies have presented growth and yield models for this region, ours is unique for several reasons. First, it provides a usable model for all forested ecosystems found in the West Coast states. While past efforts have been narrower in scope, this model can be applied to any forested area in this region, from the desert pinyon-juniper forests of Death Valley to the mesic coastal redwood forests of northern California. It can be appropriately applied to forested stands of any age, stand origin (natural vs. planted), site productivity, management intensity, and species composition. Because the data for this model was gathered from a spatially unbiased plot network, these growth and yield curves can be applied to landscape-scale carbon models such as CBM, whereas curves based on “ideal,” fully stocked stands might not capture the full variation of the landscape. Additionally, this is the only model that incorporates productivity and stockability in its biomass estimates, which allows it to be flexibly applied to a wide range of scenarios.

The predictions appear to be robust to presumed differences in management intensity. Corporate forest owners commonly employ rapid replanting of cut units with improved genetic stock, herbicide of competing vegetation, and fertilization of moderate-productivity sites to maximize volume production [63, 64]. In many cases, different yield curves are used for corporate stands than for other ownerships [15, 65]. Fitting the simple model (2) by ownership class improved model fit in three major PAZs (western hemlock, white fir-grand fir, and Douglas fir). This indicates that the curves for corporately-managed stands do indeed follow a different trajectory than the curves for Forest Service-managed stands. However, once adjustments were made for stockability and productivity, fitting separate models by ownership class no longer yielded a strong improvement in model fit within the white fir-grand fir and Douglas fir zones (Fig 3). The main deviation in the western hemlock zone was a low plateau after age 50 in corporate stands, which is not functionally realistic and likely a result of a few older stands on sites unsuitable for management. When the sample was limited to stands with ages falling within the typical rotation length of the region (50 years old), fitting separate models by ownership class no longer yielded a strong improvement in model fit within the western hemlock zone. While Forest Service management may not be as intensive as corporate management, prompt planting after regeneration harvest has been standard practice for decades. We excluded stands that were thinned at some point in their development, but this should not have affected the comparison; thinning is thought to reduce biomass accumulation [66], but the effect is perhaps transitory and not always detectable [67].

Our study suggests that it may be defensible to use one biomass yield curve for each zone, regardless of ownership. This may be preferable to relying upon different curves by ownership, given the paucity of data for extrapolating the corporate curve beyond the standard rotation age for the region. For those seeking to predict forest carbon sequestration at a landscape scale, a model based on functional traits may be more robust than a model based on ownership, since the latter assumes management regimes will remain unchanged within each ownership class indefinitely. In contrast, a model based on functional traits can adapt to changes in management regime or climate. Since the model is based on stand productivity and stockability, factors that influence those metrics could be accounted for, once the magnitude of the shift in productivity or stockability was ascertained. For instance, fertilization could enhance the productivity of a given stand, and planting trees could increase the maximum canopy cover. Subsequent measurements of that stand would produce new numbers for productivity and stockability, and a new estimate could be produced with the existing curve based on the new functional traits.

Our models predicted a lower biomass density than some comparable efforts. For instance, previously published growth and yield curves for west-side (relative to the Cascade crest) Douglas fir forests in the Pacific Northwest [65] predict a live biomass density of 246.1 Mg C/ha at year 45 for non-intensively managed stands, and 286.2 Mg C/ha for intensively managed stands. In contrast, our models predict 149.0 Mg C/ha under median stockability/productivity values for the western hemlock zone in which most west-side Douglas-fir forests occur, or 189.1 Mg C/ha when using the upper quartile of stockability/productivity values for the zone. There are several possible explanations for the discrepancy. First, our models are fit to inventory data that samples a range of conditions, not experimental plot data in stands selected for uniformity and full stocking. Our models include stands that have suffered mortality from insects and disease, whereas many growth and yield models avoid stands significantly affected by such agents [33]. Meyer [29] found that on average, westside Douglas fir stands were only 80% stocked, so accounting for these unstocked and poorly stocked stands is essential for the landscape-scale deployment of growth and yield models. Second, growth and yield models are usually built from mostly pure single-species stands. While our inventory sample of the western hemlock PAZ is dominated by Douglas fir, it includes a variety of species in various mixtures, including western hemlock, red alder (*Alnus rubra*), western red cedar (*Thuja plicata*), and bigleaf maple (*Acer macrophyllum*). Finally, while growth and yield models assume rapid regeneration following a clearcut, our models include stands that are regenerating from a variety of stand-replacing events, including harvests, insect/disease outbreaks, fire, and weather events. A recent update to Smith et al. [65] that incorporated FIA data predicted a live biomass density of 153.6 Mg C/ha at year 45 for non-intensively managed stands, and 171.8 Mg C/ha for intensively managed westside Douglas-fir stands [68], similar to the values we estimate.

A similar study to develop growth and yield curves in our region suggested that extending rotations of productive west-side Douglas-fir forests would not increase carbon stores on the landscape, the opposite of our conclusion. Diaz et al. [15] used curves from growth and yield studies of intensively managed stands up to about age 50, and then extended curves to meet the 75th percentile of FIA observations from age 60–80, and near the median value of FIA observations around 100 years old, by site class. Combining results from fully-stocked undisturbed young stands with the mean of older inventoried stands in a variety of growing conditions resulted in a flattening of volume accumulation not reflected in most growth and yield curves. Prior work indicates that maximizing merchantable volume production in this forest type would occur with much longer rotations of 100 years, or more if intermediate harvest (commercial thinning) were included [69]. Establishing a consensus on the shape of growth and yield curves in this commercially- and ecologically-important forest type would likely be useful to many users. It is possible that FIA data from the 1980s and 1990s, when most industrial owners managed westside Douglas fir on 60–70 yr rotations, might help resolve some of the discrepancies. Even so, most of the young stands on Forest Service land were on accessible, productive land and received site preparation and dense planting after regeneration harvest, while older stands were of natural origin on less accessible sites, so our chronosequence could also introduce some flattening of biomass accumulation curves with age.

Application of our models in projections of alternative management should be straightforward. The plant association zone classification is well-known to forest managers in Oregon and Washington [49], is an important variable in the FVS silviculture model [70], and can be readily crosswalked to other classifications or extracted from a predicted spatial layer for the region. The productivity variable we used (MAI) can be easily calculated from site index trees using the equations in Hanson et al. [51]. The stockability variable we used (MCC) is available on all FIA plots, can be estimated independently in a field assessment, or by selecting a point on the distribution for a vegetation zone from S1 Fig in S1 File. The models described in this analysis only describe stands that are relatively undisturbed over their lifetime. Although this provides a baseline, these models do not describe forests that have had substantial proportions of biomass killed by fire or harvest. Some landscape-scale models such as CBM [16] incorporate partial biomass removal events by “rewinding” the stand age to an earlier place on the curve reflected by the stand’s post-disturbance biomass. It is unclear whether this could be an appropriate strategy for our models to handle the biomass trajectory of stands that have been thinned. There is some evidence that pre-commercial thinning of vigorous young stands, at least, has a negligible effect on live tree carbon accumulation [71]. It might be possible to build alternative biomass accumulation models from data where the severity of events are known and biomass recovery has been measured for a sufficient time. However, the concept of “stand age” becomes less well-defined in forests that experience multiple moderate-intensity disturbances (e.g., [72]). Despite the problems, stand age is a key control variable for many forest models. Growth and yield curves simplify forest dynamics by predicting the net effect of growth and mortality, which vary somewhat predictably with stand age [48]. Long-term predictions of tree growth and mortality, for example using individual tree-level models, can be sensitive to small changes in rates and lead to unrealistic results (e.g., [73, 74]). Our empirically-based growth and yield models based on the range of forest conditions found in the Pacific coast region should prove useful for evaluating projections of these and other forest ecosystem or silviculture models. The incorporation of productivity and stockability directly into yield equations could be a useful approach for developing yield models in other forest types as well.

## Supporting information

S1 File Chisholm_Cgrowthyield. (DOCX)
