# Supplementary material for: Forest carbon sequestration on the west coast, USA: Role of species, productivity, and stockability
Source: PLoS One. 2024 May 31;19(5):e0302823. doi: 10.1371/journal.pone.0302823 (PMC11142670; doi:10.1371/journal.pone.0302823)
Supplement: S1 File — (DOCX) [file pone.0302823.s001.docx]

**Supporting information for Paul J. Chisholm and Andrew N. Gray, Forest carbon sequestration on the west coast, USA: role of species, productivity, and stockability**


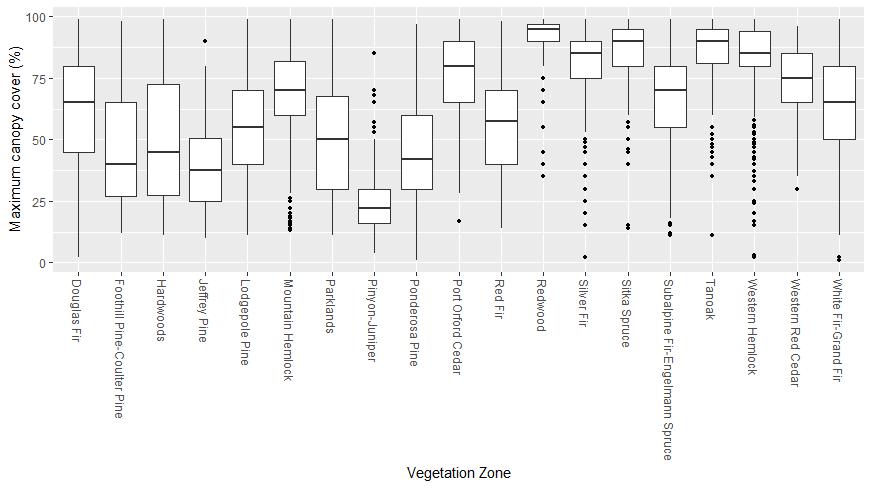


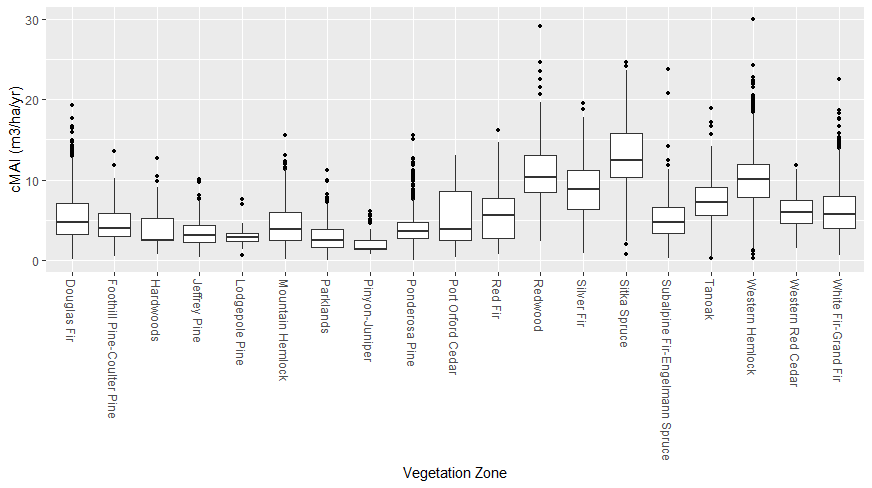


Fig S1. Distribution of values for mean annual increment at culmination (cMAI) and maximum canopy cover (% cover) by vegetation zone.

Table S1. Plot counts by current forest type.

| **Forest type** | **Sample size** |
| --- | --- |
| Pinyon-Juniper | 87 |
| Douglas Fir | 2495 |
| Ponderosa Pine | 588 |
| Western White Pine | 26 |
| Fir-Spruce-Mountain Hemlock | 1312 |
| Lodgepole Pine | 581 |
| Hemlock-Sitka Spruce | 532 |
| Western Larch | 95 |
| Redwood | 25 |
| Other Western Softwoods | 487 |
| California Mixed Conifer | 366 |
| Elm-Ash-Cottonwood | 21 |
| Aspen-Birch | 26 |
| Alder-Maple | 254 |
| Western Oak | 372 |
| Tanoak-Laurel | 180 |
| Other Hardwoods | 76 |
| Total | 7523 |

Table S2. Plot counts by ownership, within the PAZs where a comparison of USFS vs. corporate plots was possible (N ≥ 75 plots per category)

|  | **Sample size** | |
| --- | --- | --- |
| **PAZ** | **Corporate** | **USFS** |
| Douglas Fir | 75 | 475 |
| Western Hemlock | 462 | 847 |
| White Fir-Grand Fir | 120 | 1037 |

Table S3. AIC values for models fit to the Chapman-Richards equation. We modeled biomass accumulation as a function of stand age using the Chapman-Richards equation. Separate models were fit to each level of a categorical variable, either current forest type or climax forest type (i.e., vegetation zone). The model that grouped the data by vegetation zone was superior.

|  | **Model subdivisions** | |
| --- | --- | --- |
|  | **Current forest type** | **Vegetation zone** |
| **Model AIC** | 88232 | 87426 |
